# Emerging Roles of Dyslipidemia and Hyperglycemia in Diabetic Retinopathy: Molecular Mechanisms and Clinical Perspectives

**DOI:** 10.3389/fendo.2021.620045

**Published:** 2021-03-22

**Authors:** Hussain Rao, Jonathan A. Jalali, Thomas P. Johnston, Peter Koulen

**Affiliations:** ^1^ Department of Ophthalmology, School of Medicine, Vision Research Center, University of Missouri – Kansas City, Kansas City, MO, United States; ^2^ Division of Pharmacology and Pharmaceutical Sciences, School of Pharmacy, University of Missouri – Kansas City, Kansas City, MO, United States; ^3^ Department of Biomedical Sciences, School of Medicine, University of Missouri – Kansas City, Kansas City, MO, United States

**Keywords:** diabetic macular edema, diabetes mellitus, hypertriglyceridemia, lipoprotein, oxidative stress, reactive oxygen species, retina

## Abstract

Diabetic retinopathy (DR) is a significant cause of vision loss and a research subject that is constantly being explored for new mechanisms of damage and potential therapeutic options. There are many mechanisms and pathways that provide numerous options for therapeutic interventions to halt disease progression. The purpose of the present literature review is to explore both basic science research and clinical research for proposed mechanisms of damage in diabetic retinopathy to understand the role of triglyceride and cholesterol dysmetabolism in DR progression. This review delineates mechanisms of damage secondary to triglyceride and cholesterol dysmetabolism vs. mechanisms secondary to diabetes to add clarity to the pathogenesis behind each proposed mechanism. We then analyze mechanisms utilized by both triglyceride and cholesterol dysmetabolism and diabetes to elucidate the synergistic, additive, and common mechanisms of damage in diabetic retinopathy. Gathering this research adds clarity to the role dyslipidemia has in DR and an evaluation of the current peer-reviewed basic science and clinical evidence provides a basis to discern new potential therapeutic targets.

## Introduction

Diabetic retinopathy (DR) is a significant complication of diabetes mellitus (DM) characterized by ischemic microvascular disease of the retina and retinal neurodegeneration, ultimately leading to vision loss (1). Damage from DR begins as non-proliferative diabetic retinopathy (NPDR) and progresses to proliferative diabetic retinopathy (PDR). NPDR is characterized by changes in the retinal vasculature, such as increased permeability and capillary occlusion (1). Ischemia resulting from this abnormal vasculature predisposes patients to the development of angiogenesis—the hallmark of PDR (1, 2). Bleeding from the dysregulated vasculature can result in progression to diabetic macular edema (DME) (2). DME represents the main cause for vision loss in patients with DR (3). The prevalence rate of DME as a complication of DR increases with disease duration: 12% at 5–10 years after diagnosis and up to 63% after >30 years (4). It becomes imperative to further elucidate its pathophysiology, as the estimated number of people with diabetes was 422 million in 2014, with its prevalence steadily rising (1). Recent models from 2019 suggest that prevalence will increase to 578 million by 2030 and 700 million by 2050 (5). The fact that DR is a significant cause of preventable blindness worldwide (1), combined with the rapidly increasing prevalence of DM (4), demonstrates the current magnitude of this disease and the urgent clinical need for more effective therapies (2). Both metabolic syndrome and dyslipidemia have been associated with retinopathy and potentiation of DR. Examining their role and the synergistic/additive pathways between dyslipidemia and DR may prove useful in the search for novel DR therapies (1, 6, 7).

**Figure 1:**
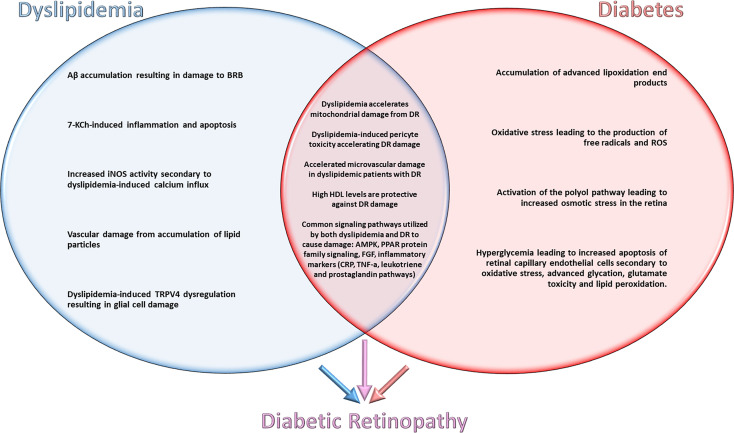
Shared and distinct mechanisms underlying dyslipidemia and diabetes contributing to the pathogenesis of diabetic retinopathy.

In order to determine how lipids impact retinal health in the context of DM, shared and distinct mechanisms underlying dyslipidemia and diabetes need to be considered (**Figure 1**). In evaluating shared mechanisms of damage between these two disease states, topics such as ROS generation, lipid peroxidation, and cellular apoptosis will receive special attention due to their deleterious effects.

Systemic therapy for glycemic control reduces the risk for developing DR and attenuates the disease progression. However, the utility of therapeutic control of abnormal lipid metabolism in DR therapy has yet to be determined (3). Dyslipidemias such as hypercholesterolemia and hypertriglyceridemia are common comorbidities of type 2 diabetes, and significantly increase the risk for microvascular complications of type 1 diabetes (8). While basic science literature describes pathways/mechanisms utilized by both hypertriglyceridemia and hypercholesterolemia, there is insufficient research on the pathogenic mechanisms of hypercholesterolemia in the absence of hypertriglyceridemia (**Figure 1**). Expanding this research could help create an understanding of pathways that play a significant role in progression of DR that do not currently have therapeutic targets. The focus of our review is to investigate these metabolic pathways and pleiotropic mechanisms to determine how both dyslipidemia and lipid-lowering therapy interact synergistically with diabetes (9). Specifically, examining the deleterious effects of dyslipidemia on the retina, hyperlipidemia has been shown to have adverse effects on health, and its role in retinal damage is also well established. This review will evaluate deleterious signaling pathways of dyslipidemia and DM identified through both basic science and clinical research in order to explore findings that could ultimately lead to the identification of novel drug targets and therapies.

## Effects of Dyslipidemia and Hypercholesterolemia on the Activity of Retinal Neurons in the Absence of Diabetes

### Preclinical Studies Providing Mechanistic and Pharmacological Evidence for Signaling Pathways Altered by Dyslipidemia Controlling the Activity of the Neural Retina

The pathways/mechanisms selected for discussion in this section are the best studied mechanisms for dyslipidemic retinal damage. Studies were selected for their evaluation of retinal damage from dyslipidemia/hypercholesterolemia with exclusion of confounding variables such as hyperglycemia. Specific pathways/mechanisms are emphasized if they are directly involved in the pathogenesis of DR and demonstrate potential as targets for pharmaceutical advancement (**Figure 1**).

#### Studies Identifying Amyloid-Beta Accumulation Secondary to High Cholesterol Diet

In rabbit models of dyslipidemia and metabolic syndrome, a high cholesterol diet increases amyloid-beta (Aβ) levels in retinal photoreceptors, inner and outer neural layers, and ganglion cell layers (9). The proposed mechanism for this damage is a compromised blood–retina–barrier (BRB). Damage to the BRB results in the leakage of plasma contents and Aβ accumulation (10). Hypoxia secondary to excess Aβ induces structural changes in retinal ganglion cells (11). These structural changes precipitate loss of functionality and apoptosis of ganglion cells.

Numerous studies have described the neurotoxic effects of Aβ peptide accumulation (12). *In vitro* models using RPE cell lines demonstrated cholesterol dysmetabolism-induced Aβ peptide accumulation (13). Decreased activity of neprilysin (NEP) and α-secretase represents an additional mechanism contributing to Aβ accumulation (13). Elevated levels of Aβ peptide in the retina ultimately results in increased production of ROS and a decrease in peroxidase activity (9). Elevated levels of ROS produce deleterious effects for retinal ganglion cells (9, 10). Additional studies have confirmed this model by pharmacologically blocking the effects of Aβ peptide (14). These pharmacological interventions demonstrated a protective role for RGCs (14).

#### Elevated Blood Levels of 7-Ketocholesterol in Diabetic Patients Inducing Inflammation

Hypercholesterolemia produces a number of oxidized byproducts that result in retinal damage. Notably, 7-ketocholesterol (7-KCh), a product formed from the oxidation of cholesterol. 7-KCh blood levels are elevated in diabetic patients (15). However, unlike Aβ peptide accumulation, elevated levels of 7-KCh have not been linked to a high cholesterol diet (15). 7-KCh activates multiple inflammatory pathways—including p38, MAP/ERK, and AKT-PKCζ-NFκB (16). Activation of the MAP/ERK pathway leads to increased oxidative stress and inflammation in retinal cells (17). 7-KCh accumulation is predominantly located in the retinal pigmented epithelium, where it can induce apoptosis secondary to the MAP/ERK signaling pathway (16). Additionally, 7-KCh induction of the NFκB pathway facilitates the expression of a number of cytokines that result in an inflammatory response (18). Notably, a primary mechanism of simvastatin’s protective role retinal ganglion cells has been attributed to the attenuation of the NFκB pathway (18).

#### Hypercholesterolemia Impairing Retinal Function *via* Inducible Nitric Oxide Synthase Pathway

In rabbit models, hypercholesteremia impairs retinal function by diminishing retinal ganglion cell density and decreasing thickness of the photoreceptor layer and inner nuclear layer (19). Destruction of these layers is correlated with an increase of inducible nitric oxide synthase (iNOS) activity (19). Increased iNOS activity results in increased oxidative damage to retinal ganglion cells. The increase iNOS activity is mediated through calcium-mediated pathways (19). Dyslipidemia plays a crucial role in stimulating this iNOS-mediated damage by increasing the intracellular calcium concentration (20). Calretinin levels, a marker for intracellular calcium levels, are elevated in the retinal neurons of animal models of dyslipidemia (20).

To further expound on the participation of lipids, we will discuss nitric oxide (NO), and the three distinct isoforms of NO synthase responsible for its synthesis: iNOS, neuronal nitric oxide synthase (nNOS), and endothelial nitric oxide synthase (eNOS). These enzymes play a critical role in endothelial dysfunction in the context of DR development and progression. It is well-established that elevated lipid levels cause endothelial dysfunction due to a marked reduction in the bioavailability of NO. Microvascular complications in DR can be detected histologically as microvascular basement membrane thickening, closures and rarefaction of capillaries, and diagnosed clinically by impaired endothelial function and eNOS uncoupling (21). Endothelial dysfunction, eNOS uncoupling, and apoptosis occur in the metabolic syndrome and type 2 DM result in microvascular damage (21). Microvascular beds located in the eyes become leaky and develop inflammation, vasoconstriction, and an overall pro-thrombotic milieu (21). In regard to hypercholesterolemia and endothelial dysfunction, markedly elevated lipids inactivate dimethylarginine dimethylaminohydrolase (DDAH). This enzyme is responsible for the metabolism of asymmetric dimethylarginine (ADMA). Continued accumulation of ADMA inhibits eNOS, which significantly limits the availability of NO to vascular endothelial cells in the eye (22, 23). This subsequently initiates endothelial dysfunction (22, 23). This cascade of events is one of many mechanisms underlying the development of endothelial dysfunction in DR. This endothelial-damage results in the breakdown of the BRB and, consequently, the exudation of serum lipids and lipoproteins (24). Interestingly, it has been estimated that 20% of retinal vascular occlusion is connected to hyperlipidemia, primarily as a result of the process described above (25).

#### Studies Examining Vascular Damage From Dyslipidemia

To date, the results from epidemiological studies focused on lipid profiles and DR are inconsistent. While some studies have determined a relationship between elevated levels of total cholesterol and LDL-cholesterol and the development and progression of DR, other studies were unable confirm these findings (26). Thus, the pathophysiologic model for the role of lipids in DR is commonly assumed to mirror the pathophysiology of atherosclerosis (26). This current model places greater emphasis on disruption of the BRB and extravasation of lipid particles in the retina, rather than attempting to simply correlate total plasma lipid and lipoprotein levels to the development of DR (26). Modified lipid particles are toxic to all retinal cell types, including endothelial cells of capillaries in the BRB. The transient disruption of the BRB ultimately leads to irreversible chronic damage. Therefore, the current consensus is that weakening of the BRB and subsequent local lipoprotein-mediated damage is more important than derangements in the overall serum lipid profile in predicting the development of DR (26).

A study from Montgomery et al. examined the retinal function of mice following the chemical induction of sustained dyslipidemia (27). Isolated dyslipidemia and its associated increase in lipid oxidation and oxidative stress led to a decline of retinal function and severely reduced b/a-wave ratios (27). The potential mechanisms for this damage include microvascular damage, disruption of neurovascular coupling, decreased apoA1 HDL and Muller cell dysfunction (27). While this retinal dysfunction did not alter the visual acuity, this study suggests that the oxidative stress from hyperlipidemia may create conditions in the retina that amplify damage from diabetes. This finding is commonly linked to retinal vascular obstruction.

#### Studies Evaluating TRPV4 Signaling Pathway as a Source of Retinal Damage

TRPV4, a nonselective cation channel that responds to cell swelling has been implicated as a potential source for retinal damage in dyslipidemia states. *In vivo* studies using mice retinal tissue suggest TRPV4 is a sensor of both physical and chemical stimuli and helps regulate the permeability of the inner retinal endothelial barrier (28, 29). An *in vivo* study of mice models by Lakk in 2017 demonstrates that dyslipidemia causes dysregulation of these TRPV4 channels and subsequent glial cell damage (29). A study using pig retinal models found that RN-1734, a specific TPRV4 inhibitor, significantly increased the ganglion cell survival, preserved retinal laminar architecture and attenuated the gliotic response (30). This study also showed that use of TRPV4 agonist resulted in extensive degenerative damage and retinal remodeling. Furthermore, *in vitro* models have demonstrated that TRPV1 and TRPV4 channel inhibition led to suppression of retinal angiogenesis (31). These results suggest an opportunity for therapeutic intervention in vaso-proliferative retinal disorders such as DR.

### Clinical Studies Providing Evidence for Dyslipidemia Pathophysiology Impairing Retina and Visual Function

Recent clinical studies of dyslipidemia resulting in impaired retinal function assist in understanding the clinical significance of the aforementioned pathways. While basic science evidence clarifies the molecular pathways of damage utilized by dyslipidemia, the clinical studies confirm these results and provide a basis for discussion of therapeutic interventions. Recent clinical studies justify expansion of research for new therapeutic targets for DR focused on hypercholesterolemia/dyslipidemia. Currently, the majority of clinical studies and therapeutic targets are focused on the attenuation of hyperglycemia.

Individuals with metabolic syndrome are significantly more likely to develop retinopathy (32). These findings were significant even in the absence of diabetes, indicating the involvement of other factors, most notably, dyslipidemia (32) and insulin resistance (33). Studies have also demonstrated components of metabolic syndrome such as increased waist circumference, higher blood pressure and low HDL to be independently associated with decreased retinal arterial caliber (34). A recent population study based in China found metabolic syndrome to be an independent risk factor for development of retinopathy in the absence of diabetes (35) (**Table 1**).

**Table 1:** Summary of clinical findings.

***1.2. Clinical studies providing evidence for dyslipidemia pathophysiology impairing retina and visual function***			
RETROSPECTIVE:	Metabolic syndrome, insulin resistance without diabetes → increased risk of retinopathy		(32–35)
	Hypertriglyceridemia in infants → increased risk of ROP		(36, 37)
**2.2 Clinical studies providing evidence for diabetes pathophysiology impairing retina and visual function**			
RETROSPECTIVE:	Increased levels of oxidative stress in the retina secondary to hyperglycemia in diabetic patients		(32, 38)
	Individuals with higher levels of aldose reductase have enhanced conversion of glucose to sorbitol, which led to an increased risk of DR development.		(39–41)
PROSPECTIVE:	Hyperglycemia increases apoptosis of retinal capillary endothelial cells secondary to oxidative stress, advanced glycation, glutamate toxicity and lipid peroxidation.		(42)
**3.2 Clinical studies providing evidence for diabetes and dyslipidemia/hypertriglyceridemia/hypercholesterolemia pathophysiologies impairing retina and visual function synergistically or additively**			
RETROSPECTIVE:	Diabetic patients with dyslipidemia had a significantly increased incidence of retinopathy when compared to diabetic patients without dyslipidemia.		(7, 14)
	A U.S. population-based study demonstrates elevated HDL to be protective against early microvascular changes and damage in diabetic patients.		
	Diabetic patients with DR exhibited greater levels of apo-A1 in their vitreous humor when compared to nondiabetic control subjects.		(43–45)
PROSPECTIVE:	In diabetic patients without DR, dyslipidemia is a predictive factor for the eventual development of retinopathy.		(46)
	Apo-A1 was found to be associated with signs of endothelial dysfunction.		(26, 47–49)
	Diabetic patients with dyslipidemia are at significantly higher risk for early retinal microvascular damage compared to diabetic patients without dyslipidemia.		(50)
**4.2 Clinical studies providing evidence for shared pathophysiologies in both dyslipidemia and diabetes contributing to the pathogenesis of DR**			
RETROSPECTIVE:	A marker of lipid peroxidation, serum malondialdehyde, was highest in poorly controlled diabetic patients, who had concurrent dyslipidemia.		(51)
	In diabetic patients with dyslipidemia, pro-inflammatory cytokines such as TNF-α and interleukins are progressively elevated as diabetes or dyslipidemia worsens.		(51)
PROSPECTIVE:	Omega-3 fatty acids decreased oxidative stress in patients with diabetes and dyslipidemia alike, suggesting an overlapping pathophysiology between these independent disease states.		(52–54)
	Several studies have suggested that statins may play a protective role in decreasing oxidative stress and potentially protecting against progression of DR.		(55–63)

Interestingly, dyslipidemia has been identified as an independent risk for a number of different retinal diseases. Premature infants frequently require total parenteral nutrition that often includes lipid emulsions. These lipid emulsions create a state of hypertriglyceridemia in these patients (36). In multiple studies, infants that develop hypertriglyceridemia demonstrated an increased rate of retinopathy of prematurity (36, 37). The proposed mechanism for this damage is that the dyslipidemic state induces metabolic dysregulation in the developing vasculature of the neural retina (37) (**Table 1**).

## Effects of Diabetes on the Activity of Retinal Neurons in the Absence of Dyslipidemia

### Preclinical Studies Providing Mechanistic and Pharmacological Evidence for Signaling Pathways Altered by Diabetes Controlling the Activity of the Neural Retina

As above, it is beneficial to explore the impact that diabetes and hyperglycemia have on retinal neurons in the absence of concurrent dyslipidemia. It is well established that the state of hyperglycemia contributes to increased oxidative stress in the diabetic retina. In addition, retinal ischemia exacerbates oxidative damage and mediates the secretion of proangiogenic factors that cause further progression of the disease. In the present section, we will explore these mechanisms to highlight elements of DR pathogenesis induced by diabetes alone in order to provide a framework to discuss the additive versus synergistic effects of diabetes and dyslipidemia in *Section Signaling Pathways and Disease Causing Mechanisms of Both Dyslipidemia and Diabetes Contributing to DR Pathogenesis* (**Figure 1**).

#### Oxidative Stress From Diabetes Resulting in Generation of Advanced Lipoxidation End Products

One way oxidative stress causes damage to cells is by the free radical mediated degradation of macromolecules, some examples of which include cellular components like membrane lipids and apolipoproteins such as LDL, VLDL, among others (64). When free radicals and ROS trigger these peroxidation cascades, the end result is the generation of advanced lipoxidation end products (ALEs). ALEs are the adducts that accumulate as a result of the non-enzymatic chemical reactions that occur in cellular constituents subjected to oxidative stress (64). This process results in the disruption of intracellular signaling, the lipoxidative damage of DNA, and the compromise of structural integrity at the level of the plasma membrane and intracellular proteins (64). This deleterious phenomenon affects cells all throughout the body, and the retina is no different (65).

In fact, studies in rat models suggest that the state of diabetes decreases the synthesis of aldehyde dehydrogenase, an enzyme that detoxifies ALEs in order to protect the retina from above mechanisms that aggravate DR, such as the disruption of cellular responses and damage to intracellular proteins (65). This is corroborated by a study conducted by Zou et al. that reports a correlation between exposure to oxidized and glycated LDL and retinal cell apoptosis (66). This phenomenon is seen both in human and diabetes-induced rat models. More specifically, diabetes seemed to increase levels of oxidized and glycated LDLs in the retina of the animals even in the absence of comorbid dyslipidemia (66). This further suggests that ALEs such as oxidized LDL may accumulate in the retina in a state of isolated hyperglycemia without necessitating a high blood lipid burden in order for deleterious apolipoproteins to cause damage to retinal neurons.

#### Studies Identifying the Protective Role of Nicotinamide Adenine Dinucleotide Phosphate Oxidase

Furthermore, retinal ischemia is critically involved in the pathophysiology of DR due to the oxidative stress that reperfusion injury inflicts on cells. Ischemia and reperfusion results in the creation of free radicals and ROS, including superoxide as a noteworthy example. The superoxide anion is particularly notable because it is a precursor to many other ROS and is produced by the enzyme nicotinamide adenine dinucleotide phosphate (NADPH) oxidase (NOX). For this reason, one might hypothesize that decreased levels of NOX should decrease the burden of oxidative stress in any cell in the body. A study tested this hypothesis on retinal neurons in mice with deletion of the NOX2 gene, which encodes a catalytic subunit of the active enzyme (67). The knockout mice with hypo-functioning NOX demonstrated reduced oxidative burden from free radical species, along with protection from neuronal death in the ganglion cell layer, when inflicted with ischemia and reperfusion in order to mimic the state of DR (67). As opposed to the NOX2 knockout mice, retinas from the wild type mice in the above study experienced markedly increased cell death apoptosis due to elevated levels of ROS and increased apoptosis signaling through phosphorylation of ERK and NFκB and subsequently allowing programmed cell death to transpire (67).

#### Studies Evaluating the Deleterious Role of the Polyol Pathway in DR Progression

The polyol pathway is pathway that results in excess glucose getting converted to sorbitol in retinal capillaries (39). This pathway mediated by aldose reductase leads to increased osmotic stress and damage to the retina and plays a crucial role in the development of retinopathy (36). This pathway has been studied both *in vivo* and *in vitro* (40, 68). An *in vitro* study found that AR inhibition led to decreased inflammatory markers, decreased AGE formation and decreased VEGF production (40). An *in vivo* study involving AR-transgenic mice found that AR blockade prevented retinal microglia (RMG) migration into nuclear layers of the retina. This decreased influx of inflammatory cells suggests a potential pharmaceutical target for preventing ocular inflammation in DR (68).

### Clinical Studies Providing Evidence for Diabetes Pathophysiology Impairing Retina and Visual Function

The adverse effects of diabetes on the activity of retinal neurons is well documented not only in the basic science experiments, but also clinical studies. While there are numerous studies examining the progression of diabetic retinopathy in patients, this section will examine those that found pathologic changes in retinal function of patients affected by diabetes without concurrent dyslipidemia. This provides a deeper understanding of the clinical implications of diabetic retinal damage prior to comparison to patients whose retinopathy is compounded with dyslipidemia (**Table 1**).

#### Studies Demonstrating the Clinical Significance of Oxidative Stress in DR Patients

The basic science literature regarding the oxidative stress that diabetes produces in the retina is corroborated with clinical research of patients. Diabetic patients have an increased level of oxidative stress placed on the retina. This level of oxidative stress is documented in patients with normal lipid levels and has also been correlated to the severity of disease (38). These conclusions are drawn by findings of elevated levels of markers for oxidative stress such as malondialdehyde (MDA) and conjugated dienes (CD) upon sampling of vitreous humor from diabetic patients (38). A study using similar markers of oxidative stress found increased levels of patients with PDR compared to NPDR (32). Their findings suggest a direct association of level of oxidative stress and severity. This higher level of oxidative stress seen in the retina of diabetic patients even in the absence of diabetes is likely related to hyperglycemic models of oxidative stress detailed in the basic science literature. A cross-sectional study evaluating the risks that individual components of metabolic syndrome play in retinopathy found hyperglycemia to have the highest odds ratio of 2.41 (2.05, 2.84) of any single component (32).The cross-sectional study from Mondal et al. evaluated patients with diabetic retinopathy to identify the biochemical pathways responsible for retinal damage (42). Their findings shed further light on the mechanisms of hyperglycemic-mediated damage. They found hyperglycemia increased apoptosis of retinal capillary endothelial cells secondary to oxidative stress, advanced glycation, glutamate toxicity and lipid peroxidation (Mondal). The demise of retinal capillary cells resulted in elevated levels of VEGF and VEGFR2, laying the framework of diabetic retinopathy (42).

#### Clinical Studies Supporting the Role of Polyol Pathway in DR

As mentioned above, the polyol pathway, also known as the sorbitol-aldose reductase pathway is considered one the major pathways specifically linking hyperglycemia with retinopathy (39). The surplus of glucose leads to activation of aldose reductase and formation of sorbitol. The accumulation of sorbitol damage the retinal capillaries through osmotic stress (40). A case control study examining 3000 patients looked at patients with and without DR and evaluated them for polymorphisms in polyol pathway genes to assess the involvement of this pathway with the development of DR (41). It was found that individuals with polymorphisms that lead to a higher level of aldose reductase had enhanced conversion of glucose to sorbitol which lead to increased risk of DR (41).

## Effects of Diabetes and Dyslipidemia on the Activity of Retinal Neurons: Synergistic or Additive?

### Preclinical Studies Providing Mechanistic and Pharmacological Evidence for Synergism in signaling Pathways Altered by Both Diabetes and Dyslipidemia Controlling the Activity of the Neural Retina

As previously discussed, there are common mechanisms of damage that dyslipidemia and hypercholesterolemia activate throughout the body. There is also evidence highlighting certain pathways of damage seen during isolation of either these disease states. In order to evaluate the therapeutic targets for diabetic retinopathy, it is imperative to review synergistic mechanisms of dyslipidemia and diabetes in the development and progression of DR. An examination of the basic science literature published on this topic reveals extensive evidence for synergistic pathways of damage in retinal neurons (16) (**Figure 1**).

#### Studies Identifying Mitochondrial Damage Secondary to Dyslipidemia

Studies employing mouse models of type 1 and type 2 diabetes suggested that dyslipidemic states potentiated the deleterious effects of diabetes on retinal neurons. Specifically, one study noted that an increase in mitochondrial damage in secondary to a dyslipidemic state resulted in quicker apoptosis of retinal capillary cells, accelerating microvascular destruction and retinal damage from diabetes (16). This evidence suggests that controlled lipid levels in pre-diabetic patients can decrease the occurrence and progression of DR.

#### Studies Assessing Oxidative Stress and Cytokine Expression Due to LDL

A study that developed new insight into the pathophysiology of diabetic retinopathy by administering oxidized LDL (ox-LDL) and ox-LDL immune complexes into human diabetic retinas post-mortem, using immunohistochemical staining for IgG in order to correlate its presence to proportional cytotoxic effects on retinal pericytes (69). Retinal pericyte cytotoxicity was determined by measuring the pericytes’ viability, receptor expression, apoptosis, endoplasmic reticulum (ER) stress and oxidative stress, and cytokine secretion; through analysis of all of these metrics, it was concluded that increased ox-LDL immune complexes predict the development of DR *via* induction of apoptosis, presence of oxidative stress and ER stress, and enhanced secretion of inflammatory cytokines (69). The study showed how retinas that displayed IHC staining for both IgG and ox-LDL had proportional severity of retinopathy, further elucidating a unique pathogenic mechanism of diabetic retinopathy which could potentially offer new targets for pharmaceutical intervention in diabetic retinas (69).

### Clinical Studies Providing Evidence for Diabetes and Dyslipidemia Pathophysiologies Impairing Retina and Visual Function Synergistically or Additively

To effectively evaluate the role that the intertwining pathophysiologies diabetes and dyslipidemia/hypertriglyceridemia have in DR, it is paramount to examine the clinical studies that evaluate patients with both pathologies. The clinical studies help delineate dyslipidemia as an effect modifier vs potential confounder the in patients DR and highlight an accelerated progression of disease in these patients. These clinical studies also provide a rationale for therapies targeting dyslipidemia can provide a clinical significant impact (**Table 1**).

Examining a national health database of Taiwan, diabetic patients with dyslipidemia had a statistically significant increase in the incidence of retinopathy when compared to diabetic patients without dyslipidemia (7). Adjusted hazard ratios for the development of NPDR and DME in dyslipidemic diabetes patients were 1.77 (CI= 1.63–1.92) and 2.34 (CI= 1.24–4.41), respectively. These significant differences support the notion of dyslipidemia potentiating damage in DR (14).

In patients without DR, studies have also found concurrent dyslipidemia to be a predictive factor for the eventual development of retinopathy. Diabetic patients with no clinical evidence of DR, have significantly lower VD (vascular density) size and FAZ (foveolar avascular zone) size than healthy patients. A study evaluating these early subclinical retinal changes in diabetics found dyslipidemia and high LDL-c to be the most significant risk factors for early retino-vascular damage (46). A recent study in JAMA Ophthalmology also supports the notion of accelerated synergistic damage in diabetes and dyslipidemia. Using OCT to evaluate structural properties of the superficial and deep vascular plexus, dyslipidemia was a significant risk factor for early retinal microvascular changes and damage with an odds ratio of 9.82 (95% CI: 6.92–11.23) (50). An additional measure that supports this correlation is the ratio of apo-A/apo-B levels. Apo-A is associated with HDL and apo-B with LDL. AN increased apo-B/apo-A ratio is correlated with the severity of DR and the apo-B/apo-A ratio represents a potential biomarker to predict damage (47). Given that apo-A1 has anti-inflammatory and anti-oxidant properties and that apo-B is associated with pro-inflammatory lipoproteins, there is consequently a positive association of the apo-B to apo-A1 ratio DR disease severity (26). This correlation is further supported by the finding that apo-A1 augments endothelial reactivity in small diameter arteries of the periphery (48). Similarly, apo-A1, and to a smaller degree apo-B levels were associated with signs of endothelial dysfunction, such as acetylcholine-induced responses of the skin microvasculature, retinal arteriolar vasodilatation induced by flicker-light, and tortuosity of retinal arterioles (49). Interestingly, these observations were made in patients without formal DR diagnosis, suggesting that these apolipoproteins indicate microvascular dysfunction, and that the association between apolipoproteins and DR is a much more robust predictor than the association between traditional serum lipid levels (*i.e.*, a conventional serum lipid profile) and DR.

The apo-B/apo-A ratio’s utility in predicting retinal damage is not only based on the damage secondary to elevated LDL but also the lack of protective HDL. A population-based study of 11,265 patients from U.S communities found elevated HDL to be protective against early microvascular changes and damage (32). Interestingly, this protective role was no longer present in people without diabetes. This suggests that not only do high LDL levels potentiate retinal damage in DR, but also, high levels of HDL can be specifically protective against progression of DR.

With regard to the role of apolipoproteins and DR discussed above, different apolipoproteins appear to have different contributions to the overall pathogenesis of DR. One study by Kawai et al. demonstrated increased levels of apo-A1 in the tear fluid of DR patients when compared to patients with DM but without DR. Importantly, it was determined that no apo-A1 was present in the tears of healthy control subjects (43). In a study examining levels of apo-A1 in the vitreous, diabetic patients with DR exhibited much greater levels of apo-A1 in their vitreous humor when compared to nondiabetic control subjects (44). Additionally, the expression of apo-A1 was much greater in the retinas of diabetics without clinically apparent DR when compared to nondiabetic controls (45). Similar to traditional mechanisms associated with the development of cardiac atherosclerosis, additional results to date support the hypothesis that apo-A1 is possibly involved with reverse cholesterol transport, which, in the case of the eye, involves HDL-stimulated efflux of lipids from the retinal pigment epithelium (RPE) potentially limiting light-induced toxicity (70, 71). Important to the pathogenesis of DR, apo-A1 has been determined to be a scavenger of ROS based on the findings of genetic apo-A1 deficiency in humans (72–74).

## Signaling Pathways and Disease Causing Mechanisms of Both Dyslipidemia and Diabetes Contributing to DR Pathogenesis

### Preclinical Studies Providing Mechanistic and Pharmacological Evidence for Shared and Parallel Signaling Pathways in Both Dyslipidemia and Diabetes Contributing to the Pathogenesis of DR

The signaling pathways/mechanisms of disease discussed in this section are common to both dyslipidemia and diabetes. A deeper understanding of the specific signaling pathways that result in damage to the retina and aid in pharmaceutical development and therapies (**Figure 1**).

#### Studies Evaluating 5’-Adenosine Monophosphate-Activated Protein Kinase in DR Pathophysiology

Several preclinical studies have identified specific molecular targets shared by the disease processes of both diabetes and dyslipidemia as relevant to both pathogenesis and disease progression; one such target is 5′-adenosine monophosphate (AMP)-activated protein kinase (AMPK). This molecule is activated within hepatocytes by metformin, a drug used to treat diabetes, resulting in both increased glucose utilization and favorable lipid profiles by lowering triglycerides and VLDL (75). *In vitro* models or rat retinal cells, AMPK-activating compounds decrease apoptosis in rat retinal Müller cells (76).

In mouse models, AMPK has been associated with improving insulin sensitivity (77, 78) and ameliorating dyslipidemia (77–80) through its regulation of lipid and glucose metabolism (81). AMPK-activating compounds reduce diabetes-induced retinal inflammation (82), demonstrating the interconnectedness of diabetes, dyslipidemia, and DR.

#### Studies Examining PPAR Protein Family Signaling in DR

PPARβ/δ signaling positively affects the lipid profile by increasing HDL cholesterol and lowering LDL cholesterol (83). Hydroxymethylglutaryl (HMG)-CoA reductase inhibitors, or statins, represent another pharmacotherapy whose pleiotropic effects offer insights into shared mechanisms of pathophysiology between dyslipidemia and diabetes. This highly efficacious lipid-lowering drug class inhibits the rate-limiting step of cholesterol synthesis (84). The pleiotropic anti-diabetes and antioxidant effects of statins (85), however, allow insights into the interconnectedness of mechanisms of both dyslipidemia and DM. Retinal microvascular endothelial cells (RMECs) treated with simvastatin demonstrated an upregulated peroxisome proliferator–activated receptor γ coactivator 1α (PGC-1α), an endogenous molecule which activates PPARγ (86). Upregulation of the PGC-1α pathway decreased generation of ROS, which contribute to DR pathogenesis by exposing both pericytes and endothelial cells of the retinal vasculature to oxidative stress thereby contributing to vascular degeneration (86). Decreased nicotinamide adenine dinucleotide phosphate (NADPH) oxidase and poly (ADP-ribose) polymerase (PARP) activity also contributed to this reduced generation of ROS (86). Furthermore, *in vivo* administration of simvastatin reduced ROS-mediated apoptosis of both pericytes and vascular endothelial cells in rats and attenuated apoptosis of bovine retinal endothelial cells *in vitro* by decreasing p38 mitogen-activated protein kinase (MAPK) activity, modulating PARP signaling, along with decreasing vascular endothelial growth factor (VEGF) expression and vascular permeability (86). Pharmacological control of the PPARγ pathway and its regulators by both TZDs and statins, respectively used for treating diabetes and dyslipidemia, illustrates the molecular interplay among pathways involved in the pathogenesis and therapy of these two disease states. Furthermore, it illustrates their mutual connections to key mediators in DR pathogenesis such as elevated ROS production and apoptosis of retinal neurons and cellular components of the retinal vasculature.

Simvastatin administration in RMEC models generated increased nitric oxide (NO) release resulting in vascular repair through induction of Akt signaling and activation of eNOS (86). In addition, *in vivo*, rosuvastatin increases Akt phosphorylation, thereby activating systemic insulin sensitivity (38); this further illustrates that diabetes and dyslipidemias share a range of common molecular targets.

Within the peroxisome-proliferator-activated receptor (PPAR) protein family of transcription factors that regulate lipid metabolism, PPARα is activated by fibrates, a class of lipid-lowering drugs (87). PPARβ/δ contributes to AMPK signaling in both dyslipidemia and DM (88), as PPARβ/δ increases AMPK levels in mouse liver (89), and synergistically activates gene expression alongside AMPK in mouse skeletal muscle, resulting in a cooperative activity that augments glucose utilization (88). Furthermore, PPARβ/δ specifically activates the AMPK pathway itself in mouse skeletal muscle, preventing ER stress-associated inflammation and insulin resistance (90). ER stress results in apoptotic cell death (91), and ER stress can be attenuated by the protective effect of AMPK activity resulting in reduced apoptosis during DR pathogenesis (92). Potential mechanistic connections among obesity, insulin resistance, and dyslipidemia resulting in ER stress (93), provide a strong rationale that such epigenetic phenomena common to dyslipidemia and diabetes can be targeted mechanistically and pharmacologically by activating AMPK.

In addition, PPARβ/δ plays a unique role in each disease state independent of AMPK activation. In mouse models of diabetes, PPARβ/δ increases insulin sensitivity through increased glucose catabolism, shunting glucose into lipogenesis pathways and increasing fatty acid oxidation in skeletal muscle (94).

Protective properties of PPARs were tested in an ex vivo model of ototoxicity using gentamicin to induce ROS production, lipid peroxidation, and apoptosis in cultured explants of the mouse organ of Corti (95). Administration of fibrates and TZDs in order to activate both PPARα and PPARγ, respectively conferred protection and attenuated apoptosis of hair cells resulting from ROS and lipid peroxide insults (95). It has been established in literature that apoptosis, free radical insult, and lipid peroxidation all contribute to disease progression in DR (92); the above study is encouraging in cementing PPAR’s cytoprotective effects throughout various disease states.

#### Studies Identifying Lipid Peroxidation as a Mechanism of Cell Damage Common to Both Hyperglycemia and Dyslipidemia

Lipid peroxidation has been identified as a hallmark of disease development and progression in both dyslipidemia and hyperglycemia, resulting in oxidative stress in retinas of diabetic rats, which contain double the amount of lipid peroxides than retinas of control rats (96). Further evidence of the inextricable linkage between chronically elevated glucose levels and dyslipidemia stems from the findings that when blood cholesterol, such as LDL, is heavily glycosylated secondary to high blood sugars, it is more likely to be oxidized when it extravasates from blood vessels (97). Moreover, distinct, disease-relevant lipid species, such as highly oxidized LDL, induce oxidative stress in the retina resulting in mitochondrial dysfunction, apoptosis and autophagy (97). When human retinal capillary pericytes (HRCP), which are critical for a functioning blood-retina barrier, are exposed to highly oxidized-glycated low-density lipoprotein (HOG-LDL), apoptosis of these pericytes resulting from HOG-LDL exposure appears to be independent of MAPK activity (98). This is therapeutically significant as increased MAPK activity results in increased apoptosis of retinal endothelial cells, a molecular pathway that is responsive to simvastatin treatment (86), as discussed above. This suggests that multiple mechanisms and signaling pathways related to oxidative stress affect retinal cell types and their function. However, both glucotoxicity and lipotoxicity aggravate diabetic retinopathy through activation of NADPH oxidase and ROS-mediated mitochondrial damage in the affected retina (99).

#### Studies Identifying a Beneficial Role for Fibroblast Growth Factor in Both Hyperglycemia and Dyslipidemia Pathologies

A critical signaling molecule at the intersection of the two pathologies is fibroblast growth factor (FGF) 21. When administered to mice, it reduces plasma glucose and triglycerides, while also lowering LDL, raising HDL, and increasing insulin sensitivity (100, 101). In addition to normalizing lipid profiles and insulin resistance, FGF21 also proved beneficial in diabetic nephropathy by improving lipid metabolism in the kidney as well as ameliorating oxidative stress (102). Systemic knockout of the FGF21 gene in mice with diabetic nephropathy resulted in more severe kidney damage, while administration of FGF21 to mice with the same pathology attenuated the nephropathy phenotype due to decreased renal lipid accumulation and oxidative stress (103). Dosing with FGF21 promoted also a small, but significant weight loss in rodents (100). Conversely, obese mice displayed resistance to FGF21 signaling (104), providing additional support for its role in controlling both diabetes and obesity (100).

#### Studies Evaluating Various Inflammatory Markers as a Common Marker of Damage

After induction of dyslipidemia and subsequently of diabetes in rats to assess the degree of oxidative stress conferred by each disease mechanism, markers of inflammation and oxidative stress were assessed (52). While in dyslipidemic rats, increased serum levels of oxidative stress (lipid peroxidation, nitric oxide, and protein carbonyl), pro-inflammatory cytokines (C-reactive protein, interleukin-1β, Monocyte chemoattractant protein-1, and tumor necrosis factor-α), and eicosanoids (prostaglandin E_2_, leukotriene B_4_, and leukotriene C_4_) were found independent of age (52), young rats that were also diabetes-induced had significantly higher concentrations of oxidative stress and inflammatory markers (52). This finding supports the notion that these disease states contribute to the same disease burden systemically, especially with regard to factors such as oxidative stress and inflammation that worsen DR (52, 92).

### Clinical Studies Providing Evidence for Shared Pathophysiologies in Both Dyslipidemia and Diabetes Contributing to the Pathogenesis of DR

Examining clinical studies for evidence of shared pathophysiologies between dyslipidemia and diabetes provides clinical significance to the aforementioned pathways known to be common in both pathologies. This evidence provides clinical relevance to the common signaling pathways previously discussed and demonstrating how they result in clinically significant progression of the severity of DR.

#### Clinical Studies Evaluating Oxidative Stress/Inflammatory Markers in Patients

An observational clinical study investigating lipid peroxidation and inflammation in patients with both diabetes and dyslipidemia yielded similar findings to Acharya’s experiment in rats; both lipid peroxidation and inflammation are higher with the presence of these two disease states (51). A marker of lipid peroxidation, serum malondialdehyde (MDA), was measured in dyslipidemic and diabetic patients—while elevated in dyslipidemic patients with or without diabetes, the highest levels were found in patients with poorly controlled diabetes complicated by dyslipidemia (51). Furthermore, pro-inflammatory cytokines (interleukin -1β, interleukin -6, interleukin -8, tumor necrosis factor-α) were progressively elevated as diabetes and dyslipidemia worsened (51). Importantly, TNF-α specifically is associated with diabetic retinopathy (92), providing a basis by which diabetes and dyslipidemia could synergistically worsen DR.

Another study obtaining similar findings as that of Acharya et al (52). concluded that omega-3 fatty acids, eicosapentaenoic acid and docosahexaenoic acid, decreased oxidative stress in human test subjects with either dyslipidemia or diabetes (55). In this trial, patients were not affected by concomitant diabetes and dyslipidemia; in each leg of the study, patients had either one disease or the other. However, the fact that oxidative stress, measured by evaluating F(2) isoprostane levels in both serum and urine, was reduced by omega-3 fatty acids in each disease state is encouraging in suggesting intertwined pathophysiology between diabetes and dyslipidemia, resulting in oxidative insult throughout the body (53).

Further clinical evidence of the systemic burden of oxidative stress and lipid peroxidation placed on the body by dyslipidemia and diabetes can be elucidated through examining statins’ effects on these parameters. Different statins have shown different levels of therapeutic benefits in various trials, as evidenced by atorvastatin for example. One study found that atorvastatin decreased markers of inflammation such as CRP as well as cellular adhesion molecules, such as intercellular adhesion molecule 1 (55). Notably, adhesion molecules cause leukostasis and increased inflammation which both contribute to DR pathophysiology (92), demonstrating a potential protective mechanism on behalf of atorvastatin. However, a more recent study assessed atorvastatin’s impact on different inflammatory mediators and saw no benefit (56). One such mediator tested was total body NO production (56); the generation of NO, specifically within mitochondria, aggravates DR (92), indicating one area of DR pathogenesis in which atorvastatin may not have therapeutic impact.

In another study assessing the impact of statins on lipid peroxidation, simvastatin treatment is associated with lower levels of malondialdehyde, C-reactive protein, and paraoxonase activity in human leukocytes taken from dyslipidemic patients with T2DM, indicating that simvastatin plays a protective role in decreasing oxidative stress and reducing lipid peroxides (57).

## Dyslipidemia, Hypertriglyceridemia and Hypercholesterolemia as Targets for Drug and Therapy Development for Diabetic Retinopathy

### Preclinical Studies Providing Mechanistic and Pharmacological Evidence That Dyslipidemia, Hypertriglyceridemia and Hypercholesterolemia Represent Viable Targets for Drug and Therapy Development for Diabetic Retinopathy

Previous sections discussing the pathophysiology and intersection of dyslipidemia/hypercholesterolemia and the progression of diabetic retinopathy lay the basis of many pharmacologic therapies. This section will focus on therapies that intend to slow progression of diabetic retinopathy that focus on targeting dyslipidemia and hypercholesterolemia. First, we will examine the basic science literature regarding the signaling pathways and molecular biology underlying these therapies.

#### PGC-1a Pathway Involvement in the Production of ROS

Statin therapy results in improvement in retinal vasculature and reduction of progression of diabetic retinopathy in both laboratory models as well as clinical studies (58, 85). This beneficial effect is the cumulative effect of altering several molecular pathways. VEGF is a driving force of proliferation in retinopathy. Simvastatin decreases mitochondrial production of ROS species through attenuation of PGC-1a pathway (85). This results in a subsequent reduction in VEGF production as well as a decrease in retinal apoptosis due to blocking -38- MAPK activation (85).

A novel PPARα agonist, AVE8134, improves both lipid profiles and glucose metabolism in diabetic mice without adverse effects of increased body weight or heart weight seen with existing anti-diabetic thiazolidinedione (TZD) medication (105). Furthermore, PPARα has therapeutic benefit in rat models of diabetic retinopathy (106). A study with novel PPARα agonist Y-0452, exhibiting reduced vascular leakage, neovascularization, and retinal cell death while improving retinal function (106).

Fibrates and other PPARα agonists that alleviate complications of diabetes elicit a range of pleiotropic effects (87). Notably, PPARα activation results in reduced inflammation as fibrates and novel PPARα agonists lower interleukin (IL)-1 induced expression of C-reactive protein (CRP), a molecule associated with coronary and vascular disease, as well as with an increased systemic inflammatory state (107). Similarly, PPARγ, a transcription factor in the PPAR protein family and a target of TZDs, diabetes drugs also known as glitazones, has beneficial effects on glycemic control while simultaneously promoting cholesterol efflux from macrophages, similar to the activity of HDL cholesterol (108, 109). Furthermore, apart from being implicated in the pathophysiology of both dyslipidemia (110) and diabetes (111), PPARγ activation through its endogenous ligand 15d-PGJ2 regulates inflammation, angiogenesis, and apoptosis in the retinal pigmented endothelium (112).

#### Therapeutic Options Seeking to Mitigate Oxidative Stress

Interestingly, omega-3 fatty acids, particularly eicosapentaenoic acid (EPA) and docosahexaenoic acid (DHA), attenuated oxidative stress and inflammation in these diabetic and dyslipidemic rats (52), positioning them as a potential preventative therapy for DR (112). Furthermore, when omega-3 polyunsaturated fatty acids are given to male leptin-receptor-deficient (db/db) mice, creating a diabetic and dyslipidemic state in this mouse model, omega-3 PUFA preserved retinal function to a degree similar to the control mice (113). This is likely a result of the fact that the levels of large *n*-3 PUFAs are typically decreased in DR, especially in the later stages of DR (114), together with a reduction in the expression of the enzymes involved with PUFA synthesis (115).

The therapeutic strategies mentioned above that primarily focus on fatty acids for treating DR are based on the unique physiological properties of the human retina. For example, polyunsaturated fatty acids, which are extremely susceptible to oxidation, are present in the membranes of photoreceptor outer segments in high concentrations (25). As the retina’s oxygen consumption is the highest of any tissue and is characterized by high oxygen tension, this potentially results in a higher susceptibility of the retina to cellular damage, such as caused by hydrogen peroxide generated from lipid peroxidation (25). The therapeutic intervention with DHA and EPA described above is founded on the fact that the retina has relatively low levels of glutathione peroxidase and catalase to counteract hydrogen peroxide generation, although it should be mentioned that the retina does possess alternative antioxidant systems such as vitamin E and superoxide dismutase (116).

As previously discussed, both diabetes and dyslipidemia cause retinal damage as a result of exposure to oxidative stress, including free radical damage that ultimately leads to deleterious accumulation of ALEs. Interestingly, administering the antioxidant tempol, which acts by neutralizing the superoxide anion, into mouse retinas with DR results in a reduction in retinal cell death as well as a reduction in intraretinal oxidized LDL and glycated LDL levels (66). This provides a possible paradigm for DR therapy directed at scavenging and inactivating hyperglycemia-induced free radicals and ALEs.

### Clinical Studies Employing or Supporting the Concept That Dyslipidemia, Hypertriglyceridemia and Hypercholesterolemia Represent Viable Targets for Drug and Therapy Development for Diabetic Retinopathy

Identifying clinical studies that demonstrate viability of dyslipidemia, hypertriglyceridemia and hypercholesterolemia as targets for therapies provides the foundation for continued development in this area of research. These studies suggest that targeting these pathologies result in clinically significant change that provides an additional level of therapeutic options to treat DR patients with these coexisting pathologies. The burden of DR on visual morbidity and the high prevalence of concurrent dyslipidemia results in a necessity to explore the clinical interventions currently possible in this domain.

As previously discussed, dyslipidemia and hypercholesterolemia disrupt retinal vasculature through a wide variety of mechanisms; the clinical findings that suggest a role of targeting these factors are discussed here. First, simvastatin can significantly reduce total cholesterol and LDL-C levels. In multiple clinical studies, patients treated with simvastatin had a significant reduction in hard deposits compared to non-treated patients (58). The beneficial effect of simvastatin was underlined by improved visual acuity as well in the treated group (58). These results suggest further discussion about whether statins should be utilized in higher doses or even as primary prevention in DR management.

In a study on statin therapy and retinal blood flow, it was concluded that mean peak systolic flow of the ophthalmic artery significantly decreased in patients with both proliferative and non-proliferative diabetic retinopathy (59). After atorvastatin treatment, the mean peak systolic flow velocities in proliferative diabetic retinopathy patients’ central ophthalmic arteries decreased an appreciable amount—implicating lower cholesterol, LDL, and total triglyceride levels in healthier retinal blood flow and reduced diabetic retinal complications (60). Furthermore, a study on the effect of lipid-lowering medication using pravastatin on DR patients showed a decrease in hard exudates in the entire patient cohort, and a decrease in microaneurisms in 66% of this patient population, suggesting that statin therapy for diabetics with hyperlipidemia could play a role in preventing background retinopathy (60).

Fenofibrate when used as monotherapy has demonstrated beneficial effects on the progression of DR (59). The FIELD study found that monotherapy with fenofibrate reduced the need for laser treatment in those with DR (61). The ACCORD Lipid Study examined the role that lipids play in the progression of retinopathy and underscored this effect of fenofibrate. The study examined 1593 participants and concluded that the addition of fenofibrate therapy to simvastatin therapy resulted in slower progression of diabetic retinopathy (62). Interestingly, the FIELD study noted that the beneficial effect of fenofibrate did not appear to be related to the plasma lipid levels of their patients.

This includes one randomized controlled trial that assessed the effect of fenofibrate on patients’ need for laser phototherapy for DR. The results indicated that treatment with laser therapy was greater in the placebo group then the fenofibrate group and that in patients with pre-existing retinopathy, 2-step progression on the ETDRS (Early Treatment Diabetic Retinopathy Study) scale, determined by funduscopic imaging, was slowed drastically (3.1% of patients on fenofibrate, vs. 14.6% on placebo) (61). What is notable, however, is that plasma HDL concentrations between the fenofibrate and placebo cohorts did not seem to play a significant role in modulating the need for laser treatment; the authors suggest perhaps intra-retinal lipid transport may differ from serum lipid concentrations in the context of eye health (61). While the beneficial effect of fenofibrate, a lipid-lowering agent, is abundantly clear in the results of this study (n = 9,795), the mechanism by which it exerts this effect (whether anti-apoptotic, anti-inflammatory, or anti-oxidative) still remains elusive (61). In another large, randomized trial (n = 10,251), it was concluded through multi-drug therapy that intensive combination treatment to reduce dyslipidemia was effective in preventing the progression of diabetic retinopathy (62). The design of this study assessed the efficacy of combination therapy, which included administration of pharmaceutical agents for glycemic, lipid, and hypertension control; as for the intervention to control patient dyslipidemia, one group was administered a statin and a placebo, while the other received a statin alongside fenofibrate as their lipid-lowering therapy (62). Intensive treatment of dyslipidemia was more effective than intensive treatment of hyperglycemia in reducing the progression of diabetic retinopathy—furthermore, patients on both a statin and fenofibrate had a rate of progression of diabetic retinopathy of 6.5%, as opposed to 10.2% with a statin and a placebo (62). This augments the findings of the above studies, showing that while statins remain effective in standard therapy, when a second lipid-lowering agent is added—especially fenofibrate—the progression of diabetic retinopathy is kept at its lowest.

A prospective, randomized, blinded trial published in the NEJM that also tested statin therapy in the context of multi-drug intervention: in this study, patients received statins as a part of their lipid-lowering therapy, while also receiving treatment for hypertension, hyperglycemia, and cardiac morbidity in the form of renin-angiotensin system blockers, aspirin, and tight glucose regulation. Such intensive therapy reduced progression of diabetic retinopathy, and the need for laser treatment for macular edema, and blindness (n = 160) (63). Furthermore, intensive multiple-drug intervention in type 2 diabetics resulted also in reduced overall vascular complications and cardiovascular risk in these patients (63).

In addition to traditional pharmacologic lipid-lowering therapy, diet and intake of specific fatty acids can play a significant role in the progression of DR. As discussed above in ***Section 5.1***, omega 3 fatty acids have demonstrated benefit in preserving retinal function in rats with concurrent diabetes and dyslipidemia. Along this same vein, Roig-Revert et al. investigated the effect of a dietary supplement containing antioxidants and omega 3 PUFAs such as glutathione and DHA in a Mediterranean cohort in order to ascertain its effect on oxidative stress biomarkers in the retina (54). A study from Sasaki et al. examining 379 patients found that in patients with well-controlled diabetes increased intake of polyunsaturated fats resulted in a reduction in presence as well as severity of DR (63). Correspondingly, they also found that an increase in saturated fats resulted in an increased risk of presence and severity of DR (63).

## Discussion, Conclusions and Future Directions

### Various Pathway Identified in Basic Science Studies to be Translated Into Clinical Studies

The review highlights areas that can be explored in clinical studies as developments for new therapeutic targets in the treatment of DR. Dissection of the current research on this topic demonstrates mechanisms/pathways described in basic science research that has yet to be explored in clinical studies. The pharmaceutical targets discussed in this section are part of pathways that have demonstrated a potential overlap between biological and clinical feasibility.

As previously mentioned, AMPK is inhibited in DR (75). Preclinical studies have demonstrated a therapeutic role for AMPK activators in both *in vitro* and *in vivo* models (79, 80). Furthermore, AMPK activators have demonstrated to be effective for numerous other diseases with minimal overt side effects (117, 118). Examples of such therapies include metformin, aspirin and resveratrol. Clinical studies regarding AMPK activators in preventing the progression of DR are indicated to assess the feasibility of this therapy.

Oxidative stress is a central component of the damage in DR (27, 38, 64, 67). Numerous pathways and mechanisms are involved in the production of this oxidative stress and this provides additional areas for the development of novel therapies (27, 64, 67). ROS production secondary to PKC activation damages retinal capillary vessels (39). Basic science evidence provides a basis to pursue clinical trials targeting this pathway. Initial clinical trials found that ruboxistaurin, a PKCB inhibitor, to have minimal benefit in the treatment of DR, however, there are numerous isoforms of PKCB that have yet to be studied in clinical studies (39, 119).

Although the polyol pathway has been directly linked with the pathogenesis of DR, clinical trials involving aldose reductase inhibitors have had inconclusive results (120, 121). However, from the basic science perspective there are both *in vitro* and *in vivo* studies demonstrating that aldose reductase blockade/knockout lead to decreased retinal damage suggesting this pathway as a therapeutic target (46, 50). Additional clinical studies are necessary to assess the utility of targeting this pathway in the prevention of DR.

### Emerging/Ongoing Clinical Studies Researching New Diagnostic Tools/Therapies for DR

There are many clinical trials that are currently taking place to assess new diagnostic tools and therapies for treatment of DR. As diagnostic tools evolve and increase in sensitivity, there is potential for earlier intervention and greater ability to observe for progress with certain treatment modalities (122). A study currently in progress is evaluating the utility of recently developed OCT angiography for diagnosis and monitoring of DR by evaluating areas of neovascularization and non-perfusion (123).

Reducing disease progression by mitigating the effects oxidation is also being explored in clinical trials. There is an ongoing randomized, multicenter interventional study in China with 1,200 participants comparing the calcium dobesilate (a potent antioxidant) to conventional treatment to assess its utility in preventing progression of DR (124). There are multiple other studies that are also currently in progress examining the utility of combined antioxidant therapy as a means to prevent progression of DR (125–127).

Lipid lowering therapy is an area of treatment that continues to be explored in clinical trials. There is currently a multi-arm trial examined the potential benefits on lipid lowering in preventing progression of DR as well as reduction of CV risk in diabetic patients (128). The three arms of this study are evaluating: Simvastatin 40mg, Fenofibrate 200mg, and Omega 3 FA (128). As previously discussed, both the FIELD study and the ACCORD Lipid Study are two major studies that indicated a potential therapeutic role for fenofibrate for reduction of DR progression, however, further clinical trials must be performed to validate these results (61, 62, 129). A randomized interventional clinical study with 1060 patients from the University of Oxford is evaluating fenofibrate vs placebo in lowering the incidence rate of non-proliferative DR (130).

## Author Contributions

Conceptualization and design, PK. Literature review, data analyzation and writing, HR, JJ, TJ, and PK. All authors have read and agreed to the published version of the manuscript. All authors contributed to the article and approved the submitted version.

## Funding

The study presented in the present publication was supported in part by Sarah Morrison Student Research Awards (HR and JJ), the Felix and Carmen Sabates Missouri Endowed Chair in Vision Research, the Vision Research Foundation of Kansas City (PK) and a departmental challenge grant by Research to Prevent Blindness (PK and TJ) and is gratefully acknowledged.

## Conflict of Interest

The authors declare that the research was conducted in the absence of any commercial or financial relationships that could be construed as a potential conflict of interest.

## Glossary

**Table:**

7KCh	7-ketocholesterol
AGE	Advanced glycation end-products
ALE	Advanced lipoxidation end products
AR	Autosomal recessive
Aβ	Amyloid-Beta
BRB	Blood-retina-barrier
CD	Conjugated dienes
CDI	Capillary density index
DM	Diabetes Mellitus
DME	Diabetic Macular Edema
DR	Diabetic Retinopathy
ER	Endoplasmic reticulum
ERK	Extracellular-signal-regulated kinase
FAZ	Foveolar avascular zone
FD	Fractal dimension
HDL	High-density lipoprotein
IHC	Immunocytochemistry
iNOS	Inducible nitric oxide synthase
LDL	Low-density lipoprotein
MAPK	Mitogen-activated protein kinase
MDA	Malondialdehyde
NADPH	Nicotinamide adenine dinucleotide phosphate
NEP	Neprilysin
NFkB	Nuclear factor kappa-light-chain-enhancer of activated B cells
NOX	Nicotinamide adenine dinucleotide phosphate oxidase
NPDR	Non-proliferative diabetic retinopathy
PARP	Poly (ADP-ribose) polymerase (PARP)
PDR	Proliferative Diabetic Retinopathy
PGC-1α	Proliferator–activated receptor γ coactivator 1α
PPAR	Peroxisome proliferator-activated receptors
RMEC	Retinal microvascular endothelial cells
RMG	Retinal microglia
ROS	Reactive Oxygen Species
RPE	Retinal pigment epithelium
TRPV4	Transient Receptor Potential Cation Channel Subfamily V Member 4
TZD	Thiazolidinedione
VD	Vascular density
VEGF	Vascular endothelial growth factor
